# A BDS–eLoran fusion positioning method for resilient PNT under reduced satellite availability

**DOI:** 10.1038/s41598-026-43921-x

**Published:** 2026-03-13

**Authors:** Jingling Li, Huabing Wu

**Affiliations:** 1 National Time Service Center, Chinese Academy of Sciences, Xi’an, 710600 China; 2 University of Chinese Academy of Sciences, Beijing, 100049 China; 3 Key Laboratory of Time Reference and Applications, Chinese Academy of Sciences, Xi’an, 710600 China

**Keywords:** GNSS, BDS, eLoran, Fusion positioning, Resilient PNT, Engineering, Mathematics and computing

## Abstract

Satellite navigation and positioning systems have become an essential component of modern infrastructure, supporting a wide range of civil and industrial applications. However, satellite-based positioning systems are vulnerable to interference, spoofing, and equipment failures, making reliance on a single system increasingly risky. To enhance system availability and continuity, the concept of resilient Positioning, Navigation, and Timing (PNT) has therefore been proposed. This paper presents a unified BDS (BeiDou Navigation Satellite System)–eLoran (enhanced Loran) fusion framework for positioning under reduced satellite observability. The framework systematically integrates BDS and eLoran measurements across different observability conditions, ranging from full satellite availability (≥ 4 satellites) to degraded scenarios with three, two, or a single satellite. Experimental results show that incorporating eLoran measurements significantly improves the robustness and availability of BDS positioning under limited satellite visibility. In particular, when the number of available BDS satellites is insufficient for conventional GNSS positioning, eLoran-assisted fusion enables continuous positioning with meter-level horizontal accuracy under favorable geometric conditions. Overall, the proposed approach demonstrates a practical fusion strategy that supports graceful performance degradation and rapid recovery under satellite-degraded conditions, especially in maritime environments.

## Introduction

In recent years, the Global Navigation Satellite System (GNSS) has made significant advances both in terms of technology and availability^1^, becoming an indispensable infrastructure of modern society. GNSS encompasses multiple satellite navigation systems, including the United States’ GPS, Russia’s GLONASS, Europe’s Galileo system, and China’s BeiDou Navigation Satellite System (BDS). Each of these systems not only provides high-precision positioning, navigation and timing (PNT) services but also offers global coverage^2^, delivering reliable navigation information to users almost anywhere on the planet^3^.

The BeiDou Navigation Satellite System (BDS), as an integral component of GNSS, is independently developed and operated by China. Despite the significant achievements of BDS in providing positioning, navigation and timing services, it still faces certain limitations and challenges, similar to other GNSS systems. These technical and security-related issues can impact overall performance and reliability. From a security perspective, BDS is vulnerable to interference, spoofing, and cyber-attacks, which may lead to misleading positioning information and severe consequences for safety-critical applications such as transportation and defense. Moreover, space debris, remnants of spacecraft and meteoroids also pose significant threats to the safety of satellites. Given the high dependence of modern society on BDS, any system malfunction or attack could have severe impacts on critical infrastructures, including transportation, communications and finance.

To address the above issues, the concept of resilient PNT has been proposed globally^4^. Resilient PNT^5^ emphasizes the ability of a navigation system to withstand disruptions, mitigate failure impacts, and rapidly recover functionality under adverse conditions^6^.

Academician Yang Yuanxi of China emphasized that the basic concept of resilient PNT is based on integrated PNT, which optimizes the integration of multi-source PNT sensors through flexible adjustments in functional models and elastic optimizations in stochastic models, thereby generating PNT information that adapts to various complex environments, ensuring high availability, continuity and reliability^7^. The U.S. report on the new “Resilient PNT Reference Architecture” highlights that the resilient nature of PNT implies its ability to adapt to changing circumstances and to respond and recover quickly from disruptions^8^.

In order to compensate for the shortcomings of satellite systems and enhance the resilience of PNT systems, additional systems have been introduced into the navigation and positioning domain. To address situations where GNSS signals become unreliable, the 33rd Digital Avionics Systems Conference (DASC) suggested the establishment of long-wave systems as a backup to GNSS systems^9^. International consensus points to the Loran system as the most suitable backup for space-based navigation and timing systems for three primary reasons: its independence, low-frequency signals, and high power^10^.

The Loran system operates on low-frequency radio waves at 100 kHz^11^, primarily propagating via ground waves along the Earth’s surface and the lower atmosphere^12–14^. The performance of this system is influenced by several factors, with the primary factors (PF) arising from atmospheric conditions. For instance, variations in the atmosphere’s average refractive index slow down the wavefront relative to the speed of light, potentially causing positioning deviations during weather changes^15^. By monitoring factors such as atmospheric pressure, temperature, and humidity, this impact can be effectively mitigated. Secondary factors (SF) primarily arise from the conductivity of the ground medium, influencing signal strength and ground wave speed^16^. The standard conductivity of seawater is a typically used parameter to estimate the impact of SF, particularly in maritime navigation when using Loran-C. Additional secondary phase factors (ASF) are influenced by terrain, especially in complex topographical areas like mountains. Such terrain effects can introduce system errors, significantly impacting the positioning accuracy of the Loran system^17–19^. The complexity of the terrain along with medium changes makes accurate calculations challenging^20^.

The General Lighthouse Authorities (GLA) of the United Kingdom and Ireland, among other entities, began evaluating eLoran ‘s performance in marine environments in the early 2000s. Research findings indicated that eLoran could offer better positioning accuracy (typically within 10 meters) compared to traditional Loran systems and provided reliable navigation services even when GNSS signals were unavailable^21,22^. To enhance eLorans accuracy, the GLA supported research into ASF correction techniques^23,24^. ASF correction is crucial for the eLoran system as it compensates for delays caused by terrain and surface characteristics along the signal path. The GLA also backed the development of hybrid receivers capable of receiving both GNSS and eLoran signals^5^. These receivers can provide high-accuracy positioning when GNSS signals are available and automatically switch to the eLoran system during GNSS outages.

As far back as 1975, Foy^25^ proposed using Taylor series estimation to derive a least squares solution from a set of simultaneously linearized algebraic equations. This method can be applied to solve many typical multi-measurements mixed-mode position-location problems encountered in navigation applications. Frank^26^ proposed the concept, theoretical analysis and demonstration of a GPS/Loran-C hybrid system based on general pseudorange processing techniques, and suggested that this system could meet the requirements for the next-generation sole means of air navigation in the United States. This idea provides valuable insights into future GNSS and Loran integration. Offermans, GWA et al.^27^ suggested that the existing Loran-C and Chayka infrastructure could be modified to serve as a backup and enhancement system for GNSS. In the event of GNSS signal disruption, a highly calibrated Loran-C/Chayka system could potentially take over navigation functions.

Chris Hide et al.^28^ proposed an integrated GPS, Loran-C and INS system for land navigation applications, in which GPS is used to estimate Loran-C signal errors when GPS measurements are available. However, in this method, GPS is only used to correct ASF errors in the Loran signal and it cannot achieve true system integration. Similar approaches, such as those discussed in^29,30^, also use GNSS to correct Loran errors. Song et al.^31^ proposed an integration algorithm for GPS/ eLoran systems but did not provide specific methods for ASF estimation. Most existing GNSS–eLoran integration studies^32–34^ still implicitly assume the availability of four or more satellites and primarily focus on performance enhancement under nominal conditions.

Therefore, this study focuses on BDS–eLoran fusion positioning under varying satellite availability, with particular emphasis on scenarios involving fewer than four satellites, which are insufficiently addressed in existing studies. Unlike conventional GNSS–eLoran integration approaches that assume full satellite visibility, the proposed framework provides a unified positioning solution applicable across one- to four-satellite configurations, including full availability (≥ 4 satellites), three-, two-, and single-satellite cases. By explicitly modeling satellite availability as a varying observability condition rather than a failure mode, the proposed design enables continuous positioning with graceful performance degradation under satellite-degraded environments.

## Method for integrated positioning using BDS/eLoran

From a methodological perspective, the core challenge addressed in this study lies in maintaining positioning solvability when the system observability degrades due to insufficient satellite measurements.

When the situation becomes worse and there are fewer than four available satellites, it is not possible to use a single BDS for positioning. In this case, eLoran signals can be introduced along with BDS signals to achieve positioning.

Long-wave positioning and timing accuracy are essentially limited by clock synchronization errors, calibration errors of transmission and reception equipment delays, along with other system errors that can be pre-measured and calibrated^35–37^. However, the errors introduced during signal propagation, such as PF, SF and ASF, must be corrected through calculations or other methods. The total error in long-wave signals can be expressed as:

1 $${T_d}=PF+SF+ASF+\delta$$

where *δ* represents system errors other than PF, SF and ASF.

PF is caused by atmospheric conditions and is mainly related to the atmospheric refractive index. It can be calculated using the following formula:

2 $$PF=nd/c$$

where n represents the refractive index of air, and c represents the speed of light.

SF is primarily influenced by the conductivity of the ground medium, represented by the ground wave attenuation factor *W*_*g*_:

3 $$SF=\frac{1}{\omega }\arg {W_g}$$

where ω represents the angular frequency.

When using BDS for ASF correction, the receiver’s position, considered the true position, is determined using BDS satellite ephemeris and pseudorange information. The geodesic distance from the receiver to the Loran transmission station can be calculated using the Vincenty formula, which is treated as the true distance *ρ* of the Loran signal. The relationship between the time it takes for the Loran signal to reach the receiver *ρ*_*c*_ and *ρ* is given by:

4 $$\frac{{{\rho _c}}}{c}=\frac{\rho }{c}+PF+SF+ASF+\delta$$

where *c* represents the speed of light. ASF can thus be expressed as:

5 $$ASF=\frac{{{\rho _c}}}{c} - \frac{\rho }{c} - PF - SF - \delta$$

Since the signal also contains random noise and other irreducible quantities, the ASF signal magnitude may fluctuate significantly. Therefore, Kalman filtering is applied to smooth the signal.

In this study, ASF correction values are derived using short-term observations collected at the experimental site. A 24-hour observation dataset is used to estimate the ASF delay by comparing the BDS-derived geodetic distance with the measured eLoran propagation time. The estimated ASF values are then smoothed using a Kalman filtering scheme to mitigate measurement noise and short-term propagation fluctuations.

It should be noted that the focus of this study is the BDS–eLoran fusion positioning framework rather than the detailed modeling of ASF itself. More comprehensive investigations of ASF prediction and long-term modeling can be found in previous studies such as Hargreaves et al.^24^.

Subsequently, the geodesic distance from the receiver to the Loran transmitter can be calculated using the Vincenty formula, which can be considered the true distance of the Loran signal. The pseudorange equation can be represented as:

6 $${\rho _i}=L({\varphi _i},{\lambda _i},\varphi ,\lambda )+{t_u} \times c+{\delta _i}$$

where, *ρ*_*i*_ is the pseudorange from the receiver to the ith positioning station, L is the actual distance from the receiver to the *i*th positioning station, ${t_u}$ is the transmission time delay, *c* is the speed of light, and ${\delta _i}$ represents other errors. The geodesic distance L is calculated using the Vincenty formula^38^.

The integration of eLoran and BDS signals provides additional measurements that can compensate for the lack of sufficient satellite signals, thereby enabling accurate localization even under adverse conditions.

The integrated positioning equations can be written as:

8 $$\left\{ \begin{gathered} {\rho _{si}}=\sqrt {{{({x_{si}} - {x_r})}^2}+{{({y_{si}} - {y_r})}^2}+{{({z_{si}} - {z_r})}^2}} +c \cdot \Delta {t_s}+{\varepsilon _{si}} \hfill \\ {\rho _{li}}=L({x_{li}},{y_{li}},{z_{li}},{x_r},{y_r},{z_r})+c \cdot \Delta {t_l}+{\varepsilon _{li}} \hfill \\ \end{gathered} \right.$$

where *ρ*_s_ represent the pseudoranges measured by the receiver to the satellites; *ρ*_l_ is the pseudorange for eLoran; (*x*_*si*_, *y*_*si*_, *z*_*si*_) represent the positions of the BDS satellites and the eLoran transmitting station; (*x*_r_, *y*_r_, *z*_r_) represent the positions of the receiver; Δ*t*_s_ represents the clock bias of the BDS satellites; Δ*t*_l_ represents the clock bias of the eLoran; *c* is the speed of light; and ε represents other errors.

Considering the different time delays between the eLoran signal reception channel and the BDS signal reception channel, an additional BDS receiver is required to achieve time synchronization with the eLoran signals.

Taking the partial derivatives of the system of equations with respect to *x*, *y*, and *z* yields the differential equations:

9 $$\left[ {\begin{array}{*{20}{c}} {\Delta {\rho _{s1}}} \\ {\Delta {\rho _{s2}}} \\ \vdots \\ {\Delta {\rho _{l1}}} \end{array}} \right]=\left[ {\begin{array}{*{20}{c}} {\frac{{x_{r}^{{}} - {x_{s1}}}}{{d_{1}^{{}}}}}&{\frac{{y_{r}^{{}} - {y_{s1}}}}{{d_{1}^{{}}}}}&{\frac{{z_{r}^{{}} - {z_{s1}}}}{{d_{1}^{{}}}}}&{\begin{array}{*{20}{c}} 1&0 \end{array}} \\ {\frac{{x_{r}^{{}} - {x_{s2}}}}{{d_{2}^{{}}}}}&{\frac{{y_{r}^{{}} - {y_{s2}}}}{{d_{2}^{{}}}}}&{\frac{{z_{r}^{{}} - {z_{s2}}}}{{d_{2}^{{}}}}}&{\begin{array}{*{20}{c}} 1&0 \end{array}} \\ \vdots & \vdots & \vdots &{\begin{array}{*{20}{c}} \vdots & \vdots \end{array}} \\ {\frac{{\partial L}}{{\partial x}}}&{\frac{{\partial L}}{{\partial y}}}&{\frac{{\partial L}}{{\partial z}}}&{\begin{array}{*{20}{c}} 0&1 \end{array}} \end{array}} \right]\left[ {\begin{array}{*{20}{c}} {\Delta {x_r}} \\ {\Delta {y_r}} \\ {\begin{array}{*{20}{c}} {\Delta {z_r}} \\ {c \cdot \Delta {t_s}} \end{array}} \\ {c \cdot \Delta {t_l}} \end{array}} \right]$$

where d refers to the distance between two points in space.

10 $$\left\{ {\begin{array}{*{20}{c}} {\frac{{\partial L}}{{\partial x}}=\frac{{\partial \rho }}{{\partial \varphi }} \cdot \frac{{\partial \varphi }}{{\partial x}}+\frac{{\partial \rho }}{{\partial \lambda }} \cdot \frac{{\partial \lambda }}{{\partial x}}} \\ {\frac{{\partial L}}{{\partial y}}=\frac{{\partial \rho }}{{\partial \varphi }} \cdot \frac{{\partial \varphi }}{{\partial y}}+\frac{{\partial \rho }}{{\partial \lambda }} \cdot \frac{{\partial \lambda }}{{\partial y}}} \\ {\frac{{\partial L}}{{\partial z}}=\frac{{\partial \rho }}{{\partial \varphi }} \cdot \frac{{\partial \varphi }}{{\partial z}}+\frac{{\partial \rho }}{{\partial \lambda }} \cdot \frac{{\partial \lambda }}{{\partial z}}} \end{array}} \right.$$

The partial derivatives of *ρ* with respect to *φ* and *λ* can be found as described in^32^. Then, solving for the partial derivatives of *φ* and *λ* with respect to *x*, *y*, and *z* will be performed as follows:

11 $$\left\{ {\begin{array}{*{20}{c}} {\frac{{\partial \varphi }}{{\partial x}}=\frac{{(p - {e_2}a{{\cos }^3}\theta )(3{e_1}b{{\sin }^2}\theta \cos \theta \frac{{\partial \theta }}{{\partial x}}) - (z+{e_1}b{{\sin }^3}\theta )(\frac{x}{p}+3{e_2}a{{\cos }^2}\theta \sin \theta \frac{{\partial \theta }}{{\partial x}})}}{{{{(p - {e_2}a{{\cos }^3}\theta )}^2}+{{(z+{e_1}b{{\sin }^3}\theta )}^2}}}} \\ {\frac{{\partial \varphi }}{{\partial y}}=\frac{{(p - {e_2}a{{\cos }^3}\theta )(3{e_1}b{{\sin }^2}\theta \cos \theta \frac{{\partial \theta }}{{\partial y}}) - (z+{e_1}b{{\sin }^3}\theta )(\frac{x}{p}+3{e_2}a{{\cos }^2}\theta \sin \theta \frac{{\partial \theta }}{{\partial y}})}}{{{{(p - {e_2}a{{\cos }^3}\theta )}^2}+{{(z+{e_1}b{{\sin }^3}\theta )}^2}}}} \\ {\frac{{\partial \varphi }}{{\partial z}}=\frac{{(p - {e_2}a{{\cos }^3}\theta )(3{e_1}b{{\sin }^2}\theta \cos \theta \frac{{\partial \theta }}{{\partial z}}) - (z+{e_1}b{{\sin }^3}\theta )(\frac{x}{p}+3{e_2}a{{\cos }^2}\theta \sin \theta \frac{{\partial \theta }}{{\partial z}})}}{{{{(p - {e_2}a{{\cos }^3}\theta )}^2}+{{(z+{e_1}b{{\sin }^3}\theta )}^2}}}} \end{array}} \right.$$

12 $$\left\{ {\begin{array}{*{20}{c}} {\frac{{\partial \lambda }}{{\partial x}}=\frac{{ - y}}{{{x^2}+{y^2}}}} \\ {\frac{{\partial \lambda }}{{\partial y}}=\frac{x}{{{x^2}+{y^2}}}} \\ {\frac{{\partial \lambda }}{{\partial z}}=0} \end{array}} \right.$$

13 $${e_1}=\frac{{{a^2} - {b^2}}}{{{b^2}}}$$

14 $$p=\sqrt {{x^2}+{y^2}}$$

15 $$\theta =\arctan \left( {\frac{{z \cdot a}}{{p \cdot b}}} \right)$$

To improve positioning accuracy, a dynamic weight matrix *W* is introduced. Unlike conventional GNSS–eLoran integration methods that rely on fixed or heuristic weighting schemes, the proposed approach employs a geometry- and error-aware adaptive weighting strategy, in which measurement weights are dynamically adjusted according to real-time satellite geometry (HDOP) and propagation-induced uncertainties (ASF residuals). This design enables the fusion process to automatically adapt to evolving measurement quality and observability conditions. The weight of the *i*-th measurement source is given by:

16 $$W=\left[ {\begin{array}{*{20}{c}} {{w_1}}&0&0&0 \\ 0&{{w_2}}&0&0 \\ 0&0&{{w_3}}&0 \\ 0&0&0&{{w_4}} \end{array}} \right],$$

17 $${w_i}=\frac{1}{{\sigma _{i}^{2} \cdot HDOP_{i}^{2} \cdot (1+{\varepsilon _{ASF}})}}$$

where ${\sigma}_{i}^{2}$ is the pseudorange noise variance, ${HDOP}_{i}$ is the horizontal dilution of precision, and ${\varepsilon}_{ASF}$ is the normalized ASF residual.

This formulation achieves three effects: Sources with poor geometry (high HDOP) are down weighted; Measurements with large ASF residuals are suppressed. The weights automatically adapt to changing signal conditions.

After introducing the weight matrix, the objective function is defined as:

18 $$f(r,\Delta t)={(AX - B)^T}W(AX - B),$$

Here, X= [x, y, z, $\varDelta t$] ^T^ is the vector to be solved, A is the design matrix, and B is the pseudo-range measurement vector.

By minimizing the objective function f (x, $\varDelta t$), the weighted least squares solution can be obtained:

19 $$k={({A^T}WA)^{ - 1}}{A^T}WB$$

The ASF temporal drift $\delta ASF$ and receiver clock bias $\delta t$ are estimated simultaneously through:

20 $${[\delta x,{\text{ }}\delta t,{\text{ }}\delta ASF]^T}={({A^T}WA)^{( - 1)}}{A^T}WB$$

**Fig. 1:**
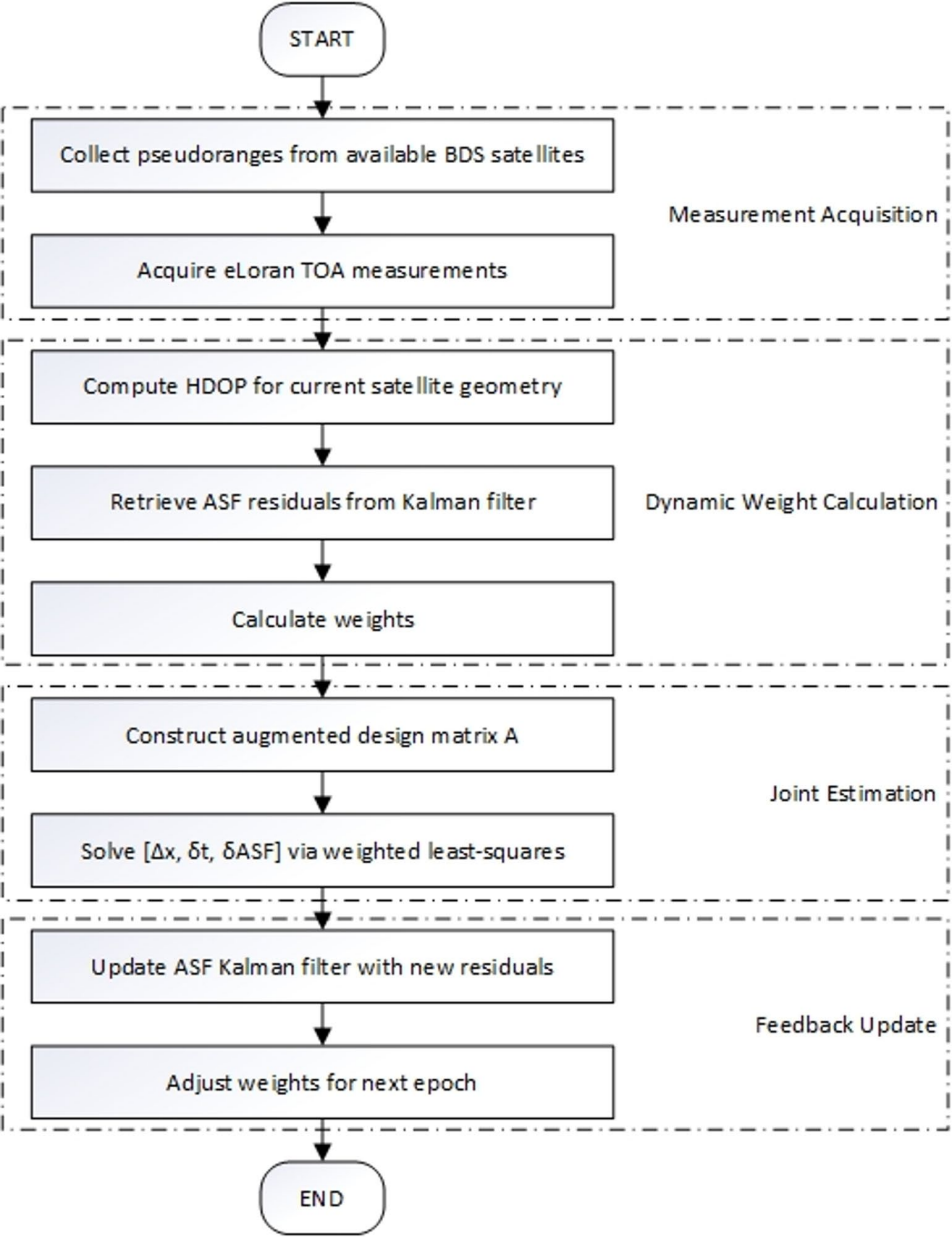
Solution flowchart of the BDS/eLoran fusion positioning algorithm.

At this point, the integrated positioning results of BDS and eLoran have been obtained, with the detailed solution process illustrated in Fig. 1.

**Fig. 2:**
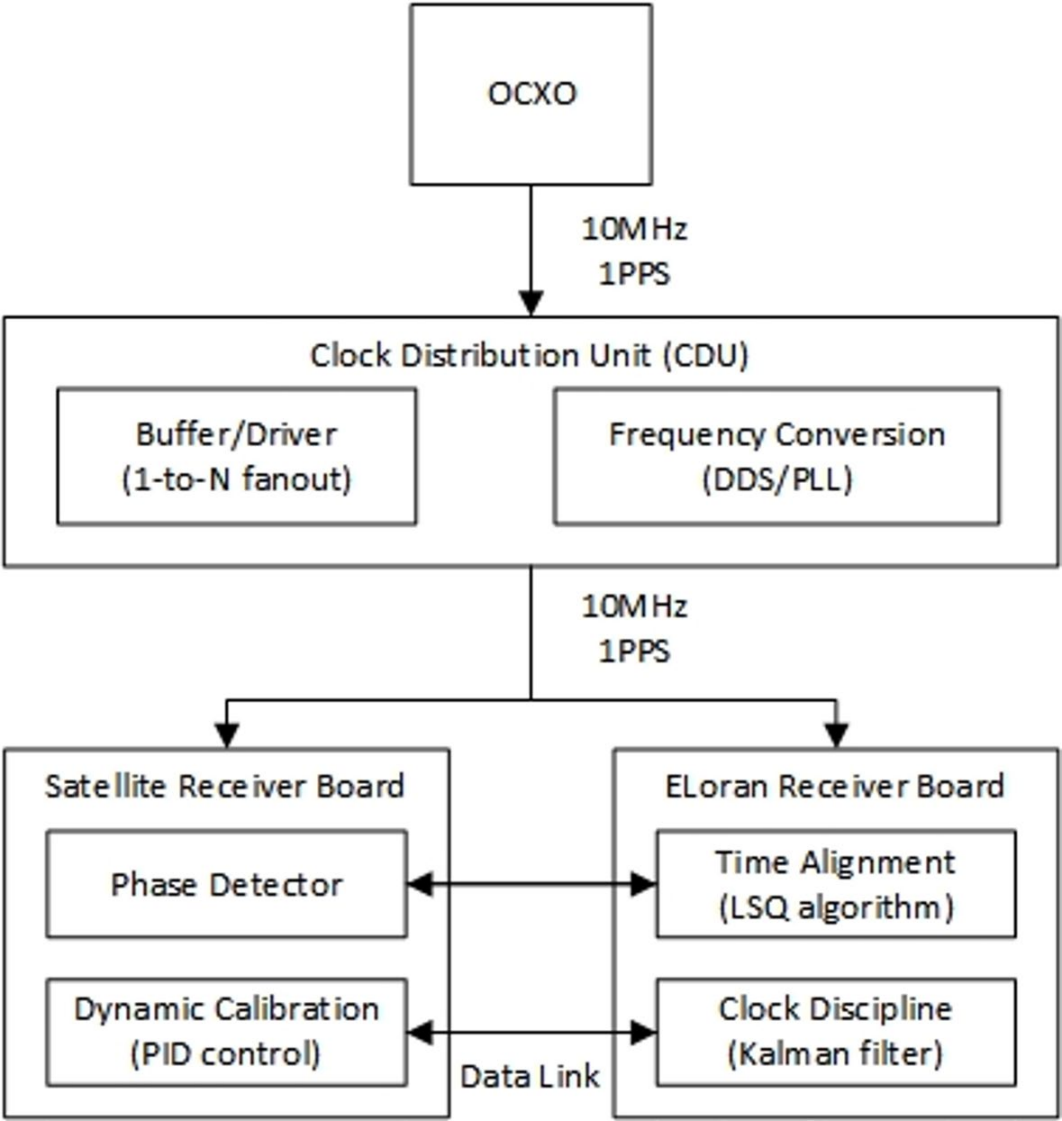
Time synchronization implementation process.

Even if enough usable satellite signals can be received, we can still integrate satellite data with eLoran for positioning. The benefits of doing so include several aspects: (1) It helps improve the HDOP value of the positioning range. (2) It may introduce higher-quality eLoran signals, thereby enhancing positioning accuracy. (3) When satellite signals are interfered with or suddenly interrupted, the eLoran system can still continuously provide PNT (Positioning, Navigation, and Timing) services (Fig. 2).

The complete set of pseudo-range equations can be expressed as:

21 $$\left\{ \begin{gathered} {\rho _{s1}}=\sqrt {{{({x_{s1}} - {x_r})}^2}+{{({y_{s1}} - {y_r})}^2}+{{({z_{s1}} - {z_r})}^2}} +c \cdot \Delta {t_s}+{\varepsilon _{s1}} \hfill \\ {\rho _{s2}}=\sqrt {{{({x_{s2}} - {x_r})}^2}+{{({y_{s2}} - {y_r})}^2}+{{({z_{s2}} - {z_r})}^2}} +c \cdot \Delta {t_s}+{\varepsilon _{s2}} \hfill \\ \begin{array}{*{20}{c}} \vdots \\ {{\rho _{sn}}=\sqrt {{{({x_{sn}} - {x_r})}^2}+{{({y_{sn}} - {y_r})}^2}+{{({z_{sn}} - {z_r})}^2}} +c \cdot \Delta {t_s}+{\varepsilon _{sn}}} \end{array} \hfill \\ {\rho _{l1}}=\sqrt {{{({x_{l1}} - {x_r})}^2}+{{({y_{l1}} - {y_r})}^2}+{{({z_{l1}} - {z_r})}^2}} +c \cdot \Delta {t_l}+{\varepsilon _{l1}} \hfill \\ {\rho _{l2}}=\sqrt {{{({x_{l2}} - {x_r})}^2}+{{({y_{l2}} - {y_r})}^2}+{{({z_{l2}} - {z_r})}^2}} +c \cdot \Delta {t_l}+{\varepsilon _{l2}} \hfill \\ {\rho _{l3}}=\sqrt {{{({x_{l3}} - {x_r})}^2}+{{({y_{l3}} - {y_r})}^2}+{{({z_{l3}} - {z_r})}^2}} +c \cdot \Delta {t_l}+{\varepsilon _{l3}} \hfill \\ \end{gathered} \right.$$

For the BDS/eLoran integrated positioning system, time synchronization between the two systems is a critical issue. A single temperature-compensated crystal oscillator (TCXO) is used to provide a common time and frequency reference for both the BDS and eLoran receivers. The oscillator drives the local clock of the BDS module, which is continuously calibrated using satellite signals to correct clock bias. Simultaneously, a disciplining circuit adjusts the oscillator frequency to match the timing of the eLoran signal. In the event of BDS signal loss, the system can rely on eLoran to maintain timing; conversely, BDS can be used to correct the accumulated timing errors of the eLoran system.

### Improvement of HDOP in the integrated BDS/eLoran system

Geometric Dilution of Precision (GDOP) is a critical parameter in satellite navigation systems, which measures how the satellite geometry affects positioning accuracy^39^.

HDOP (Horizontal Dilution of Precision) represents the impact on horizontal positioning accuracy, which focuses on the positioning accuracy in the horizontal direction (east and north). Its mathematical definition is:

22 $$HDOP=\sqrt {\sigma _{E}^{2}+\sigma _{N}^{2}}$$

where: ${\sigma}_{E}^{2}$ is the standard deviation of the eastward positioning error, and ${\sigma}_{N}^{2}$ is the standard deviation of the northward positioning error.

**Fig. 3:**
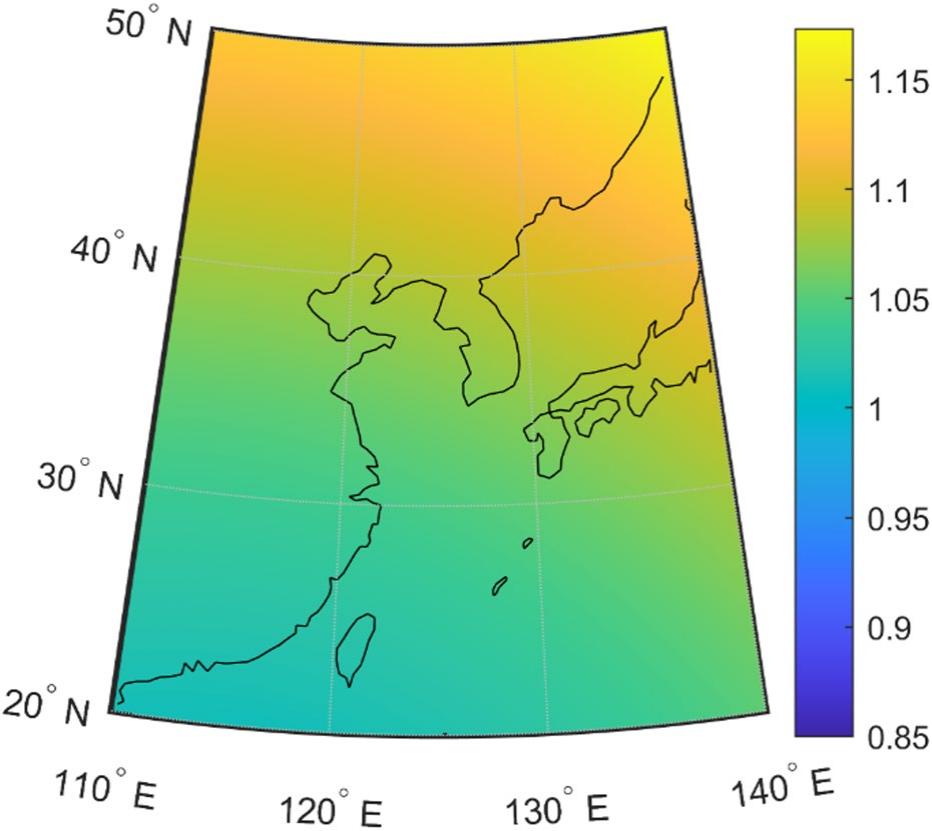
HDOP values of four satellites. The map visualization was generated using MATLAB R2023a (MathWorks, Natick, MA, USA, https://www.mathworks.com).

During the experimental phase, eight available satellites were detected, referred to as Sat1 through Sat8. Additionally, the eLoran stations were named Loran1, Loran2 and Loran3. At a particular moment, the receiver is receiving signals from three BDS satellites (Sat2, Sat6, Sat7, and Sat8). The HDOP values are shown in Fig. 3.

It can be observed that within the range of 20–50° N latitude and 110–140° E longitude, the HDOP values of the selected four satellites are all less than 2. This also ensures high positioning accuracy from the perspective of satellite geometric distribution.

Next, the HDOP performance of the BDS/eLoran integrated system will be verified. The design matrix G of the integrated system can be expressed as:

23 $$G=\left[ {\begin{array}{*{20}{c}} {\frac{{x_{r}^{{}} - {x_{s1}}}}{{r_{1}^{{}}}}}&{\frac{{y_{r}^{{}} - {y_{s1}}}}{{r_{1}^{{}}}}}&{\frac{{z_{r}^{{}} - {z_{s1}}}}{{r_{1}^{{}}}}}&1 \\ \vdots & \vdots & \vdots & \vdots \\ {\frac{{x_{r}^{{}} - {x_{sn}}}}{{r_{n}^{{}}}}}&{\frac{{y_{r}^{{}} - {y_{sn}}}}{{r_{n}^{{}}}}}&{\frac{{z_{r}^{{}} - {z_{sn}}}}{{r_{n}^{{}}}}}&1 \\ {\begin{array}{*{20}{c}} {\frac{{\partial {L_1}}}{{\partial x}}} \\ \vdots \\ {\frac{{\partial {L_m}}}{{\partial x}}} \end{array}}&{\begin{array}{*{20}{c}} {\frac{{\partial {L_1}}}{{\partial y}}} \\ \vdots \\ {\frac{{\partial {L_m}}}{{\partial y}}} \end{array}}&{\begin{array}{*{20}{c}} {\frac{{\partial {L_1}}}{{\partial z}}} \\ \vdots \\ {\frac{{\partial {L_m}}}{{\partial z}}} \end{array}}&{\begin{array}{*{20}{c}} 1 \\ \vdots \\ 1 \end{array}} \end{array}} \right]$$

Currently, eLoran systems are primarily deployed in regions with critical requirements for maritime navigation or resilient PNT services, such as Northern Europe (e.g., the United Kingdom and Norway), East Asia (e.g., China and South Korea), and North America. In China, the North China Loran Chain (NCLC) comprises several transmitting stations, including Rongcheng (37.2°N, 122.5°E), Xuancheng (30.9°N, 118.8°E), and Helong (42.5°N, 129.0°E). This network provides ground wave coverage across the Yellow Sea and East China Sea, with a typical transmission range of approximately 1,000 to 1,500 km under standard atmospheric propagation conditions.

**Fig. 4:**
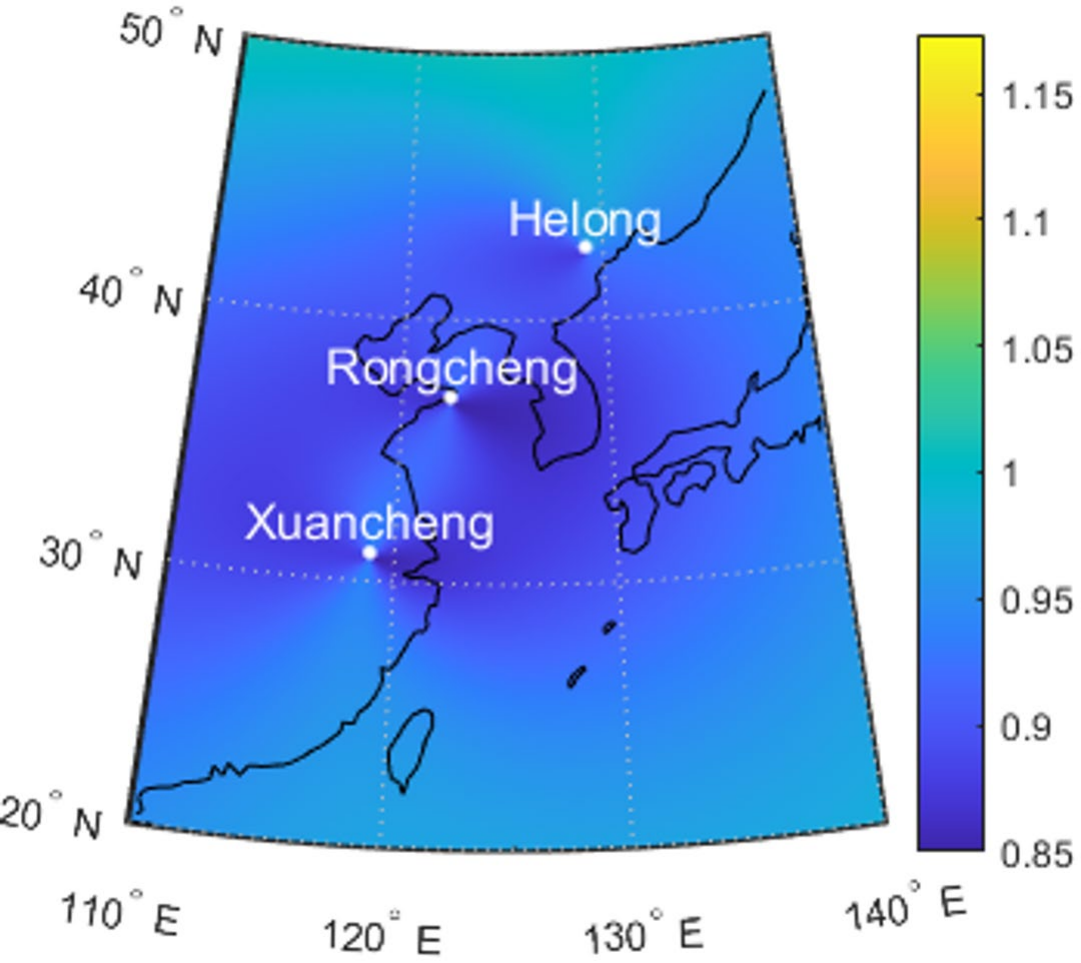
HDOP values of four satellites and three eLoran stations (Rongcheng, Xuancheng and Helong). The map visualization was generated using MATLAB R2023a (MathWorks, Natick, MA, USA, https://www.mathworks.com).

Figure 4 shows that the HDOP values resulting from the integration of four satellites with three eLoran stations are less than 1 within the eastern sea area of China. This indicates that there are already good positioning conditions in this region.

### Experimental setup and equipment description

The experimental platform consists of a dual-system BeiDou/eLoran integrated receiver developed by the National Time Service Center of the Chinese Academy of Sciences. The BeiDou module supports reception of different frequency signals, including B1I, with a sampling interval of 1 s; the eLoran receiver operates at a carrier frequency of 100 kHz and a group repetition interval of 7430 µs.

Both receivers use a temperature-compensated crystal oscillator (TCXO) as a common frequency reference and are equipped with a time interval counter to ensure time synchronization. The eLoran antenna is a vertically polarized active whip antenna optimized for low-frequency reception; the BeiDou antenna is a geodetic-grade GNSS antenna with multipath suppression capabilities.

Measurement data was continuously recorded for more than 24 h at each test point.

## Results and discussion

To investigate the positioning capability of integrating eLoran stations with BDS, an experiment was conducted in the eastern maritime area of China using BDS receivers and eLoran receivers that had completed time synchronization.

Our test site lies within the overlapping coverage of NCLC, ensuring signal-to-noise ratios (SNR) > 10 dB during the experiment. This aligns with ITU-R P.684-7 recommendations for maritime eLoran reception.

**Fig. 5:**
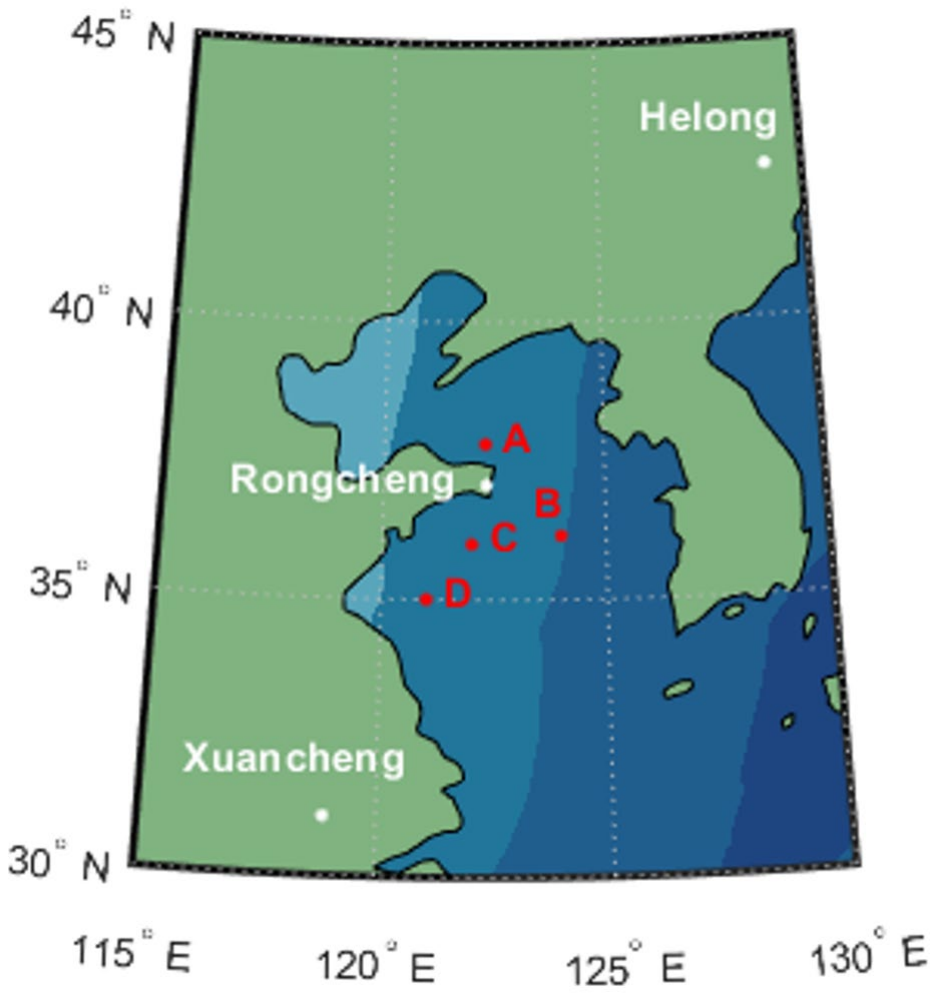
Distribution map of eLoran stations and test positions. The map was generated using MATLAB R2018b (MathWorks, Natick, MA, USA, https://www.mathworks.com).

First, the positioning performance of eLoran was verified at a fixed test point. Three eLoran stations were located in Rongcheng, Shandong; Xuancheng, Anhui; and Helong, Jilin. These stations belong to the North China Loran Chain, with a group repetition interval of 7,430. The experimental locations and the distribution of the eLoran stations are shown in Fig. 5. In addition, the locations of the four-test points A, B, C, and D are marked in Fig. 5. Due to space limitations, only the experimental analysis for point B is presented in the following sections. The receiver equipment used in the experiment was provided by the National Time Service Center of the Chinese Academy of Sciences.

**Fig. 6:**
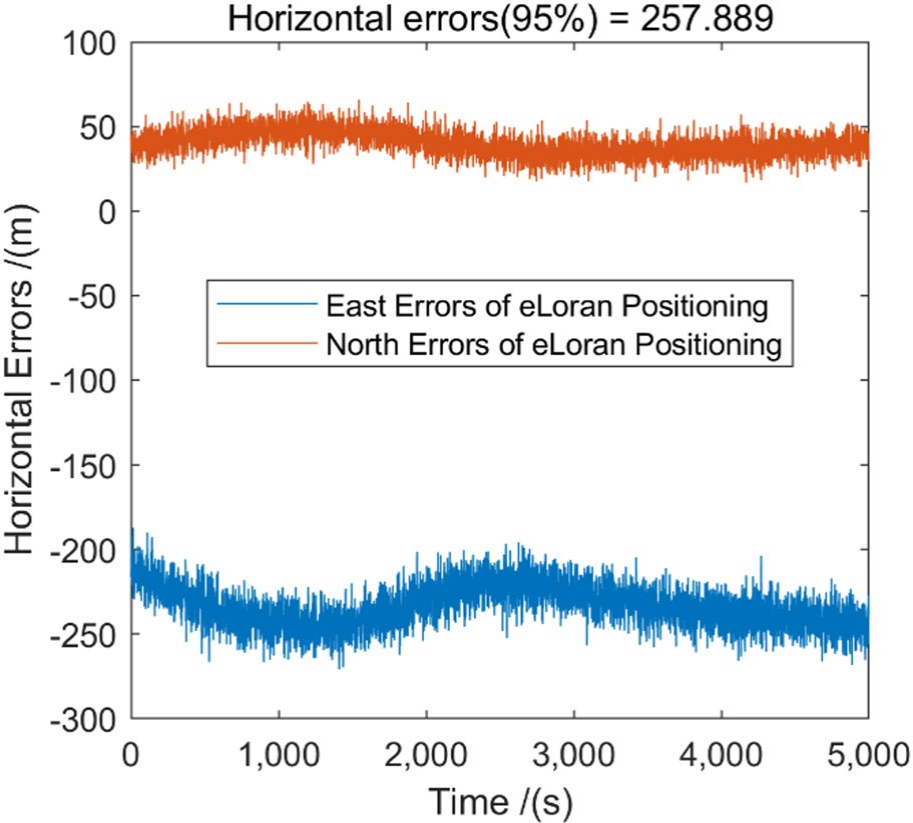
The horizontal positioning error (HPE) of eLoran without ASF correction.

Continuous 24-hour eLoran signal sampling was conducted at the test point. The average BDS positioning solution over a 24-hour period was adopted as a reference position, under the assumption of stable satellite geometry and nominal signal conditions during the observation interval.

Figure 6 illustrates the horizontal positioning errors of eLoran without ASF correction. It can be observed that due to the significant impact of ASF on signal propagation time, the positioning and timing performance of eLoran are poor when ASF is not corrected. The HPE (95%) reached 257.889 m.

**Fig. 7:**
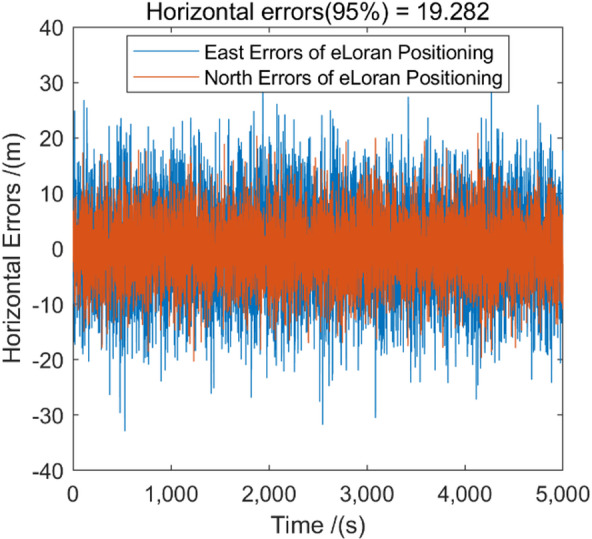
The horizontal positioning error of eLoran after ASF correction.

**Fig. 8:**
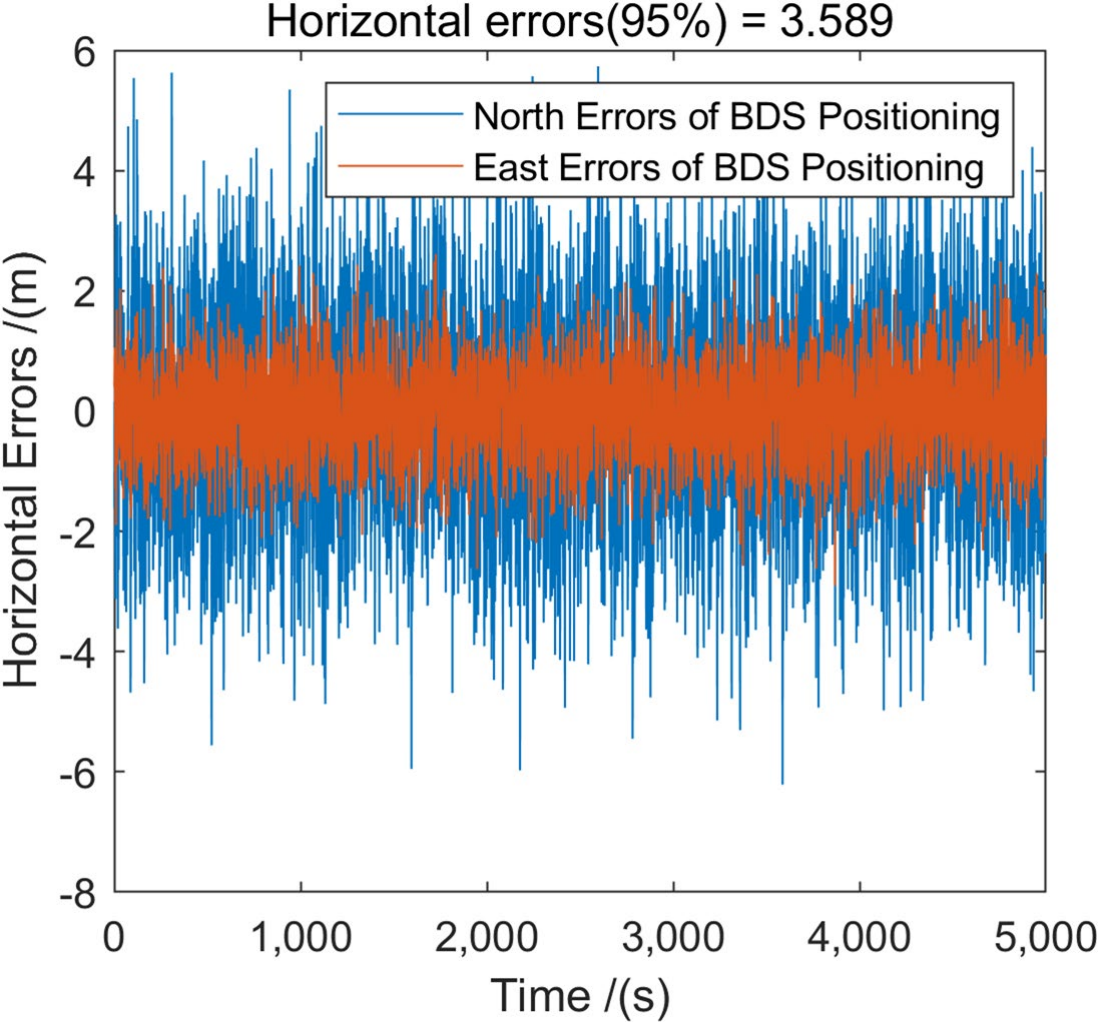
The BDS positioning error at the fixed test point.

Figure 7 show the positioning errors of eLoran after ASF correction. It can be seen that by using BDS to correct the ASF of eLoran, the positioning capability of eLoran has been improved, with the HPE (95%) reaching 19.282 m, which is much closer to the positioning performance of BDS (Fig. 8). These results confirm that BDS-assisted ASF correction significantly improves the positioning performance of eLoran, providing a reliable basis for subsequent BDS–eLoran fusion.

With the high positioning and timing accuracy of the eLoran signal, the positioning and timing performance after integrating the BDS signal with the eLoran signal can be analyzed. Using a strategy where the number of satellites and eLoran stations increases from few to many, the HPE values for different fusion scenarios were verified: one satellite with three eLoran stations (with the constraint of Earth’s surface), two satellites with three eLoran stations, three satellites with three eLoran stations, and four satellites with three eLoran stations.

**Fig. 9:**
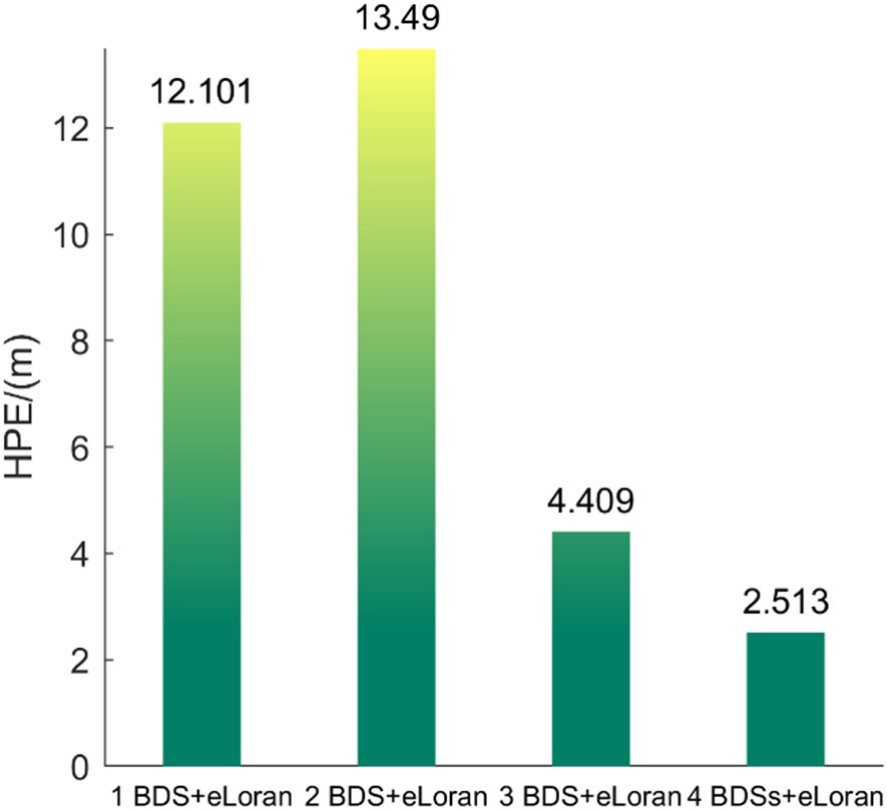
HPE of fusion positioning with different numbers of satellites and three eLoran stations.

The eLoran signal has already been pre-corrected for ASF. As shown in Fig. 9, with the increase in the number of satellites, the overall HPE tends to decrease. When one satellite is fused with three eLoran stations, the constraint of an altitude of 0 m is applied, which is equivalent to adding a satellite positioned at the Earth’s center. In the case of fusing two satellites with three eLoran stations, no constraint is applied, so the HPE values for these two configurations are relatively similar, at 12.101 m and 13.490 m, respectively. The addition of three satellites significantly reduces the HPE value to 4.409 m, and when four satellites are added, the HPE value drops below the HPE value of 2.513 m, as shown in Fig. 11 for positioning with only four satellites. The results reveal a consistent relationship between system observability, geometric strength, and fusion performance, indicating that the proposed framework degrades gracefully rather than catastrophically as satellite availability decreases.

However, it is important to note that when the number of satellites is small, especially fewer than four, the spatial distribution of satellites must be strictly controlled. Only when the spatial distribution of satellites and eLoran stations is good, meaning a low HDOP value, will the positioning results be more accurate. Furthermore, there are still some issues, including the need for a higher number of iterations and the potential for singular solutions during the calculation.

Next, the fusion positioning effect will be verified by manually turning the BDS signal channel on/off (increasing or decreasing the available satellites).

**Fig. 10:**
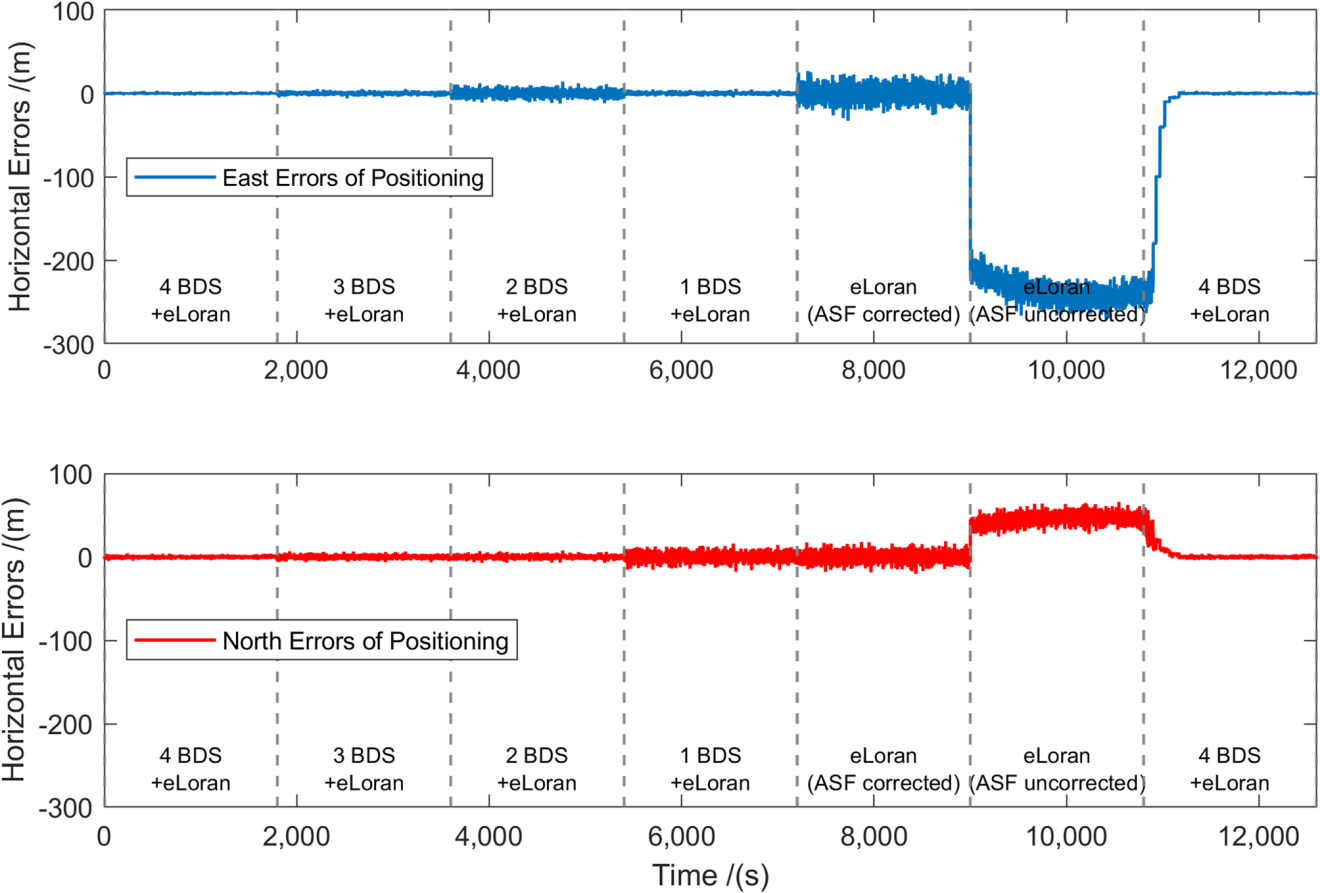
HPE of BDS and eLoran fusion positioning when adjusting the number of satellites.

The testing process was structured into seven sequential stages, each lasting 30 min. These stages correspond to progressively reduced satellite availability and recovery conditions: full fusion positioning using four BDS signals and three eLoran signals; fusion positioning with three BDS signals and three eLoran signals after disabling one BDS satellite; fusion positioning with two BDS signals and three eLoran signals after disabling an additional BDS satellite; fusion positioning with one BDS signal and three eLoran signals, with the altitude constrained to zero; eLoran-only positioning with ASF correction applied after disabling all BDS signals; eLoran-only positioning without ASF correction to simulate prolonged BDS outages without ASF map support; and full signal restoration to simulate the satellite recovery process. This altitude constraint is mathematically equivalent to introducing a pseudo-measurement that stabilizes the vertical component of the solution, thereby preserving horizontal positioning solvability under severely limited satellite geometry.

The positioning errors in the horizontal plane are divided into eastward and northward components, as shown in Fig. 10. Specifically, when the number of satellites decreases, the HPE increases, and the positioning accuracy decreases. The relative sizes of the eastward and northward error components change due to the different spatial distribution of satellites and eLoran stations, which can be attributed to the variation in HDOP values. After BDS signals are reintroduced, the HPE significantly decreases after a period of recovery, and the positioning accuracy can be restored to the initial level of fusion positioning with four satellites and eLoran.

**Table 1:** Positioning performance of BDS/eLoran at fixed test points.

Test points	HPE (95%) (m)							
	W with HDOP and ASF				W without HDOP and ASF			
	4 BDS + eLoran	3 BDS + eLoran	2 BDS + eLoran	1 BDS + eLoran	4 BDS + eLoran	3 BDS + eLoran	2 BDS + eLoran	1 BDS + eLoran
A	2.795	4.752	15.537	15.924	3.134	5.769	18.138	18.312
B	2.513	4.409	13.490	12.101	3.263	5.542	17.554	16.977
C	2.613	5.124	14.322	14.311	3.517	5.923	16.342	17.583
D	2.493	4.497	13.916	12.416	3.246	5.626	17.215	17.151

Table 1 presents the 95% HPE for four different BDS/eLoran integration configurations across the four test points. A reduction in the number of satellites generally leads to an increase in HPE. Additionally, the spatial distribution of satellites—reflected in the HDOP—also significantly influences the positioning accuracy. When comparing the configurations of 2 BDS satellites + eLoran and 1 BDS satellite + eLoran, it is observed that the latter sometimes achieves better HPE performance. This is likely because the single BDS + eLoran case includes a hypothetical satellite located at the Earth’s center, which may yield a more favorable HDOP than the configuration using two actual satellites.

By comparing the weighting matrix 𝑊 that incorporates HDOP and ASF values with the one that does not, it is observed that the inclusion of HDOP and ASF leads to a 20%–30% reduction in positioning error, indicating the effectiveness of the dynamic weighting matrix.

**Fig. 11:**
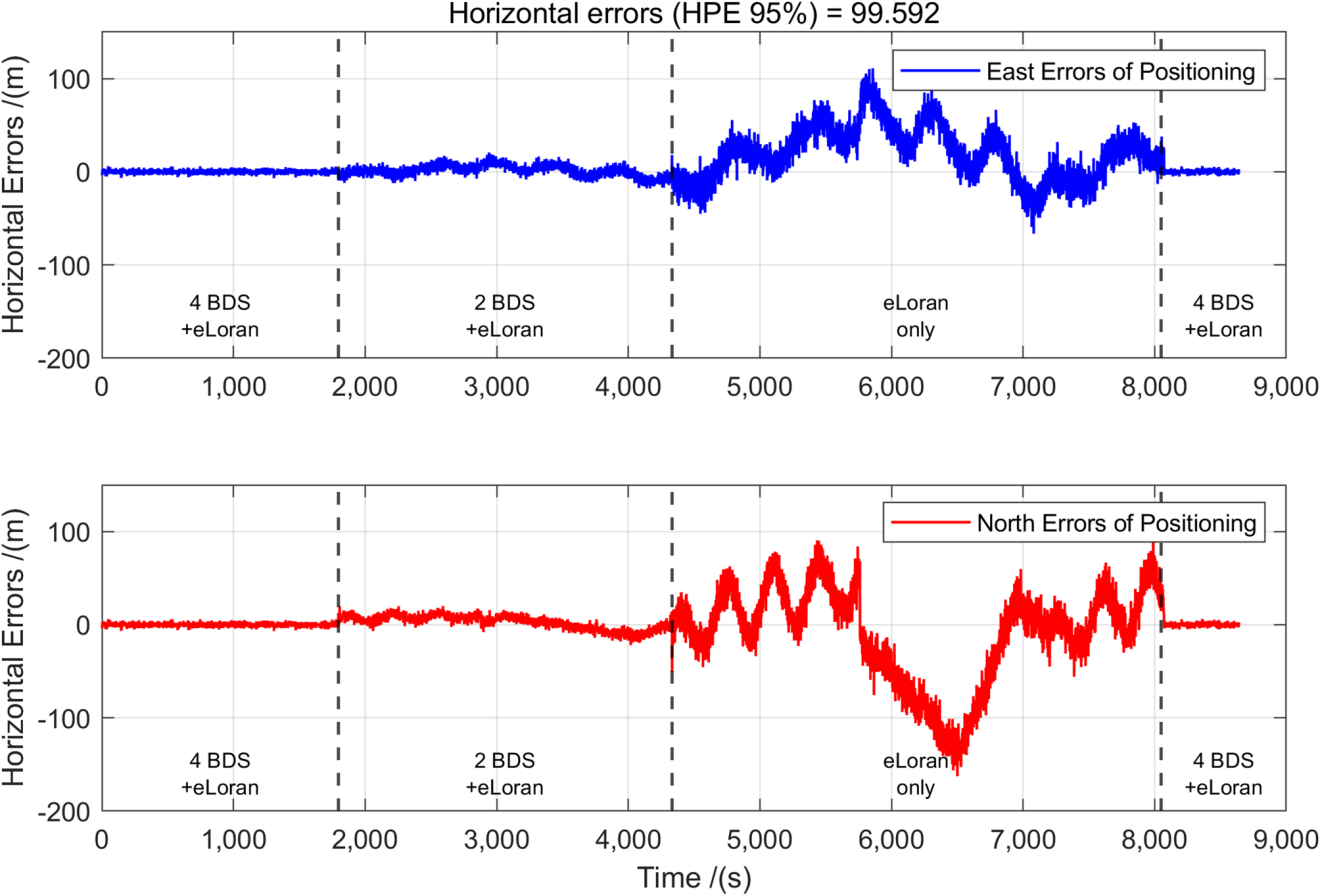
HPE of BDS and eLoran fusion positioning when adjusting the number of satellites.

To evaluate the dynamic positioning performance of the BDS/eLoran integrated system, a navigation test was conducted near Test Point B. The test was divided into four-time segments: (1) positioning using the full set of BDS satellites and eLoran, (2) positioning with only two BDS satellites and eLoran, (3) positioning with eLoran only, and (4) resumption of full BDS and eLoran positioning. The corresponding positioning errors are shown in Fig. 11.

As illustrated in the figure, during the first phase, where both full BDS and eLoran signals were available, the positioning error remained relatively low. In the second phase, when only two satellites were used, noticeable fluctuations appeared in the positioning error, with a variation range of approximately 20 m. This indicates that the reduction in satellite availability significantly degraded positioning accuracy, although it still performed better than the third phase with no satellite signals.

During the third phase, where only eLoran was used, large error spikes occurred as the vessel moved back and forth. These fluctuations are largely attributed to variations in the ASF delay. Once the BDS receiver was reactivated in the fourth phase, the positioning error quickly stabilized and returned to a constrained range.

The experimental results show that the performance of the proposed fusion system is highly dependent on the spatial geometry of the available BeiDou satellites and eLoran stations. When the HDOP is small, the system can achieve a HPE below 10 m even with only 1 satellite + 3 eLoran stations. When the HDOP is large, the dynamic weighting mechanism automatically compensates and gives priority to eLoran measurements with higher geometric stability ($1/HDO{P^2}$ term).

Our dynamic BDS–eLoran fusion framework addresses two key limitations of prior studies. First, regarding geometry adaptability, conventional GNSS solutions fail under sub-4-satellite conditions, whereas the proposed method maintains solvable positioning even with a single satellite, achieving a 95% horizontal positioning error (HPE) of 12.1 m by augmenting satellite measurements with eLoran signals and height constraints derived from terrestrial surface models. Second, in terms of error resilience, the inclusion of the ASF residual term (Eq. 17) mitigates the influence of abnormal measurements during abrupt terrain transitions, effectively suppressing outliers and reducing positioning errors.

While optimized for maritime environments, extension to terrestrial and airborne applications presents additional challenges. Urban and mountainous regions exhibit low ground conductivity (< 0.001 S/m), attenuating eLoran signals; airborne deployment above ~ 3 km is constrained by ground-wave propagation. Future work will address these limitations through terrain-aware ASF prediction using high-resolution elevation data (e.g., SRTM, LiDAR) and tightly coupled MEMS inertial measurements for terrestrial scenarios, and hybrid VIS/IR beacon systems for airborne operations.

The framework enables graceful performance degradation and rapid service recovery under reduced satellite availability: HPE remains 12–20 m with one to three satellites, compared with complete failure in conventional GNSS, and reacquisition occurs in < 2 s once satellite signals are restored, satisfying IMO recovery standards (Fig. 11). Additionally, multi-tiered service allows trading positioning accuracy for availability, e.g., ~ 19 m using eLoran-only positioning or ~ 4 m with full BDS availability.

Overall, the proposed fusion system establishes a robust baseline for resilient PNT, particularly in maritime applications, with dynamic weighting and unified sub-4-satellite processing providing a template for future multi-source PNT systems.

However, the proposed method still has several limitations. First, the current system is primarily optimized for marine and coastal environments, where eLoran ground-wave propagation is relatively stable and ASF characteristics can be effectively modeled. Consequently, its direct application to dense urban, mountainous, or high-altitude airborne scenarios is constrained by increased signal attenuation and terrain-induced propagation effects.

Second, the performance of the proposed method depends on the availability and accuracy of ASF correction information. The ASF correction employed in this study is derived from short-term observations under relatively stable propagation conditions; long-term ASF drift and seasonal variability were not explicitly investigated and may affect positioning performance during prolonged GNSS outages, particularly in regions with pronounced land–sea transitions.

Finally, experimental validation was conducted in a specific maritime area with a limited number of observation stations. Although the results demonstrate consistent performance trends, further large-scale experiments under diverse geographical and environmental conditions are required to fully assess the general applicability of the proposed framework.

## Conclusion

This study presents a unified BDS–eLoran fusion framework for positioning under sub-four-satellite conditions, extending conventional GNSS integration approaches that rely on full satellite availability. The framework employs a dynamically adaptive fusion strategy with real-time weight optimization and joint estimation of ASF temporal drift and receiver clock bias, accelerating convergence during GNSS recovery while ensuring reacquisition within 2 s.

Experimental validation in the East China Sea demonstrates gradual and predictable performance degradation as satellite availability decreases: horizontal positioning error transitions smoothly from ~ 2.5 m under full four-satellite availability to 12.1 m with a single satellite supported by three eLoran stations, avoiding abrupt positioning failure. Incorporation of ASF correction and geometry-aware optimization further enables continuous positioning during GNSS outages, highlighting the framework’s potential to enhance availability and robustness in resilient PNT applications.

Importantly, this work reframes sub-four-satellite positioning as a continuous observability spectrum within a constrained fusion framework, rather than treating reduced satellite availability as an immediate failure case. Future work will focus on extending the proposed framework to additional navigation sources, such as inertial sensors and shortwave systems, and on evaluating its performance in more complex terrestrial and mixed-environment scenarios.

## Data Availability

The data generated and analyzed during the current study are available from the corresponding author on reasonable request.
